# Bioactivities and Structure–Activity Relationships of Fusidic Acid Derivatives: A Review

**DOI:** 10.3389/fphar.2021.759220

**Published:** 2021-10-15

**Authors:** Junjun Long, Wentao Ji, Doudou Zhang, Yifei Zhu, Yi Bi

**Affiliations:** School of Pharmacy, Key Laboratory of Molecular Pharmacology and Drug Evaluation, Ministry of Education, Collaborative Innovation Center of Advanced Drug Delivery System and Biotech Drugs in Universities of Shandong, Yantai University, Yantai, China

**Keywords:** fusidic acid, biological activities, structure-activity relationship, tetracyclic triterpene, antimicrobial

## Abstract

Fusidic acid (FA) is a natural tetracyclic triterpene isolated from fungi, which is clinically used for systemic and local *staphylococcal* infections, including methicillin-resistant *Staphylococcus aureus* and coagulase-negative *staphylococci* infections. FA and its derivatives have been shown to possess a wide range of pharmacological activities, including antibacterial, antimalarial, antituberculosis, anticancer, tumor multidrug resistance reversal, anti-inflammation, antifungal, and antiviral activity *in vivo* and *in vitro*. The semisynthesis, structural modification and biological activities of FA derivatives have been extensively studied in recent years. This review summarized the biological activities and structure–activity relationship (SAR) of FA in the last two decades. This summary can prove useful information for drug exploration of FA derivatives.

## 1 Introduction

Over the past 40 years, more than half of the new chemical entities approved for the treatment of various diseases have originated from unmodified natural products, their semi-synthetic derivatives, or synthetic biological analogs (Newman and Cragg, 2020). Natural products are rich in structural types and have a wide range of biological activities, and are the main source for the discovery of new chemical entities and lead compounds (Chen et al., 2017; Agarwal et al., 2020). Thus, natural products have long been regarded as important sources in drug design, especially for drugs for cancer and infectious diseases (Brown et al., 2014; Rodrigues et al., 2016; Agarwal et al., 2020). Furthermore, almost all-important natural products, such as terpenes, alkaloids, sesquiterpenes, and sugars, can be produced by fungi (Aly et al., 2011; Singh et al., 2019).

Fusidic acid (FA) is a tetracyclic triterpenoids isolated from fungi, which was first isolated from *Fusidium coccineum* in 1960 (Godtfredsen WO. et al., 1962; Godtfredsen W. O. et al., 1962). FA binds to elongation factor G (EF-G) as an inhibitor of protein synthesis (Yamaki, 1965; Kinoshita et al., 1968). Since 1962, FA has been clinically used for systemic and local *staphylococcal* infections, including methicillin-resistant *Staphylococcus aureus* (MRSA) and coagulase-negative *staphylococci* infections (Godtfredsen WO. et al., 1962; Zhao et al., 2013). FA has been widely used throughout Europe, Australia, China, India, and other countries. There are many reasons why FA is not approved by USFDA, especially in funds and laws (Fernandes and Pereira, 2011). At present, FA is being promoted for approval in the United States market by Cempra Pharmaceuticals (ClinicalTrials.gov, 2020).

FA and its derivatives have been shown to possess a wide range of pharmacological activities, including antibacterial (Godtfredsen W. O. et al., 1962), antiparasitic (Gupta et al., 2013; Salama et al., 2013), antituberculosis (Cicek-Saydam et al., 2001), anticancer (Ni et al., 2019), tumor multidrug resistance (MDR) reversal (Guo et al., 2019), anti-inflammation (Kilic et al., 2002), antifungal (Bi et al., 2020), and antiviral activity (Liu et al., 2019) *in vivo* and *in vitro*. However, there is no review on the semisynthesis, biological activities, and structure–activity relationship (SAR) studies of FA derivatives in the last 2 decades. This review summarized the semisynthesis, modification and bioactivities of FA derivatives. The SARs of FA and its derivatives in antibacterial, antiparasitic, antituberculosis, antitumor and tumor MDR reversal were summarized. This review provides useful information for the development of FA derivatives and gives a direction for further inspiration to enrich its structures with good pharmacological activities.

## 2 The Biological Activities and Structure–Activity Relationships of FA

### 2.1 Antimicrobial Activity

#### 2.1.1 Anti-Gram-Positive Bacterial Activity

**FIGURE 1:**
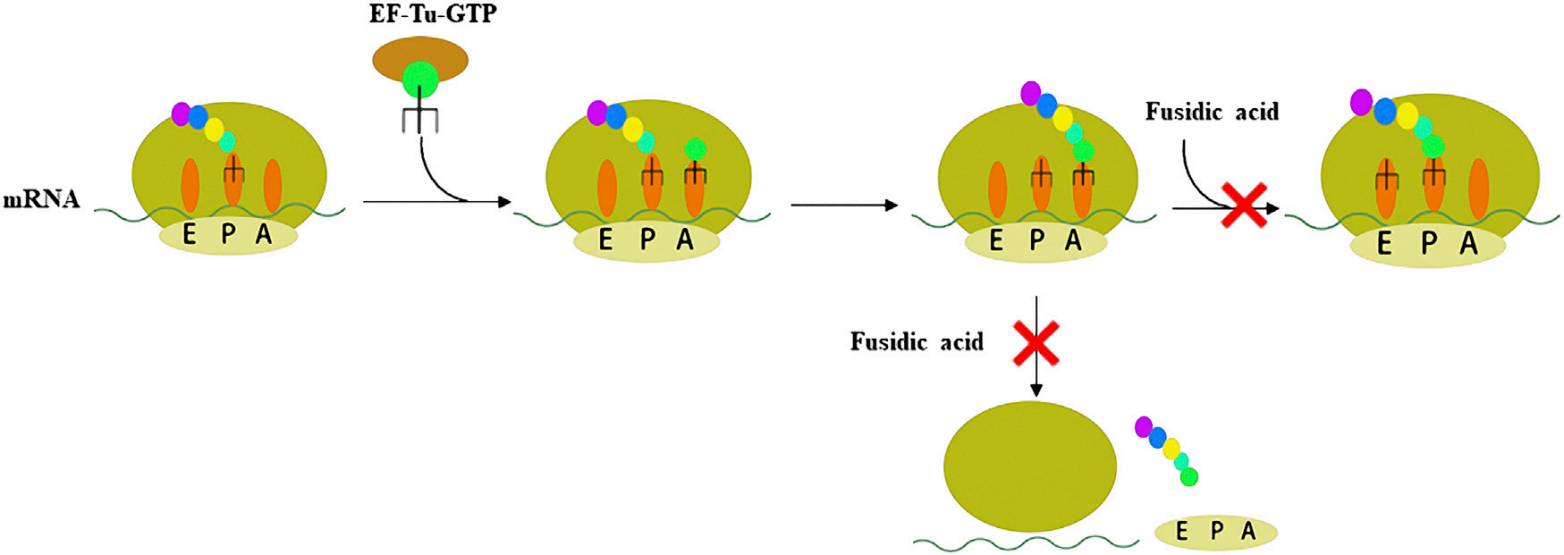
Schematic representation of the two steps of peptide synthesis that FA blocks by binding to the EF-G-GDP complex.

Resistance to antibiotic is a major obstacle to treating bacterial infection (Centers for Disease Control and Prevention, 2019). Therefore, antibiotics with novel mechanisms of action and low drug-resistance to bacteria are needed. FA acts on EF-G, which is the only antibiotic that acts on this target. There are four stages of protein synthesis in bacteria: initiation, extension, translocation, and recycling (Figure 1, Fernandes, 2016). Translocation is catalyzed by EF-G with GTPase activity. FA forms a stable complex with EF-G-GTP hydrolysate (EF-G-GDP), which causes the translocation to be blocked (Bodley et al., 1969; Belardinelli and Rodnina, 2017). Another function of EF-G is to split the terminated ribosome with the help of ribosome releasing factor, and then the next mRNA translation can occur, so FA also blocks the recycling stage (Savelsbergh et al., 2009; Wilson, 2014). In other words, FA blocks the translocation and recycling stages of protein synthesis, thereby killing bacteria through this mechanism. Additionally, FA lacks appreciable cross-resistance with other antibiotics, which is mainly attributed to the particular mechanism of action of FA (Borg et al., 2015).

According to previous metabolism studies of FA, the sodium salt of FA is well absorbed after oral administration with a bioavailability higher than 90%, and the FA binds highly and reversibly to protein (Turnidge, 1999; Still et al., 2011). Because of the high protein binding rate of FA, hyperbilirubinemia or jaundice is one of the main side effects of FA (Rieutord et al., 1995; Lapham et al., 2016). Many FA derivatives have been synthesized to develop antibiotics with better pharmacokinetic and pharmacodynamic profiles.

In 1979, Daehne et al. have synthesized more than 150 FA analogs, including modifications to the skeleton, the A, B, C, and D rings, and the side chains of FA, but the antibacterial activity of most of these derivatives was reduced or even completely abolished. The activity of a few compounds was maintained or enhanced, including derivatives with saturation of the delta-24 (25) double bond (**1**), substitution of the 16*α*-acetoxy by other groups (**2**), and conversion of the 11-OH to the corresponding ketone group (**3**) (Daehne et al., 1979).

**FIGURE 2:**
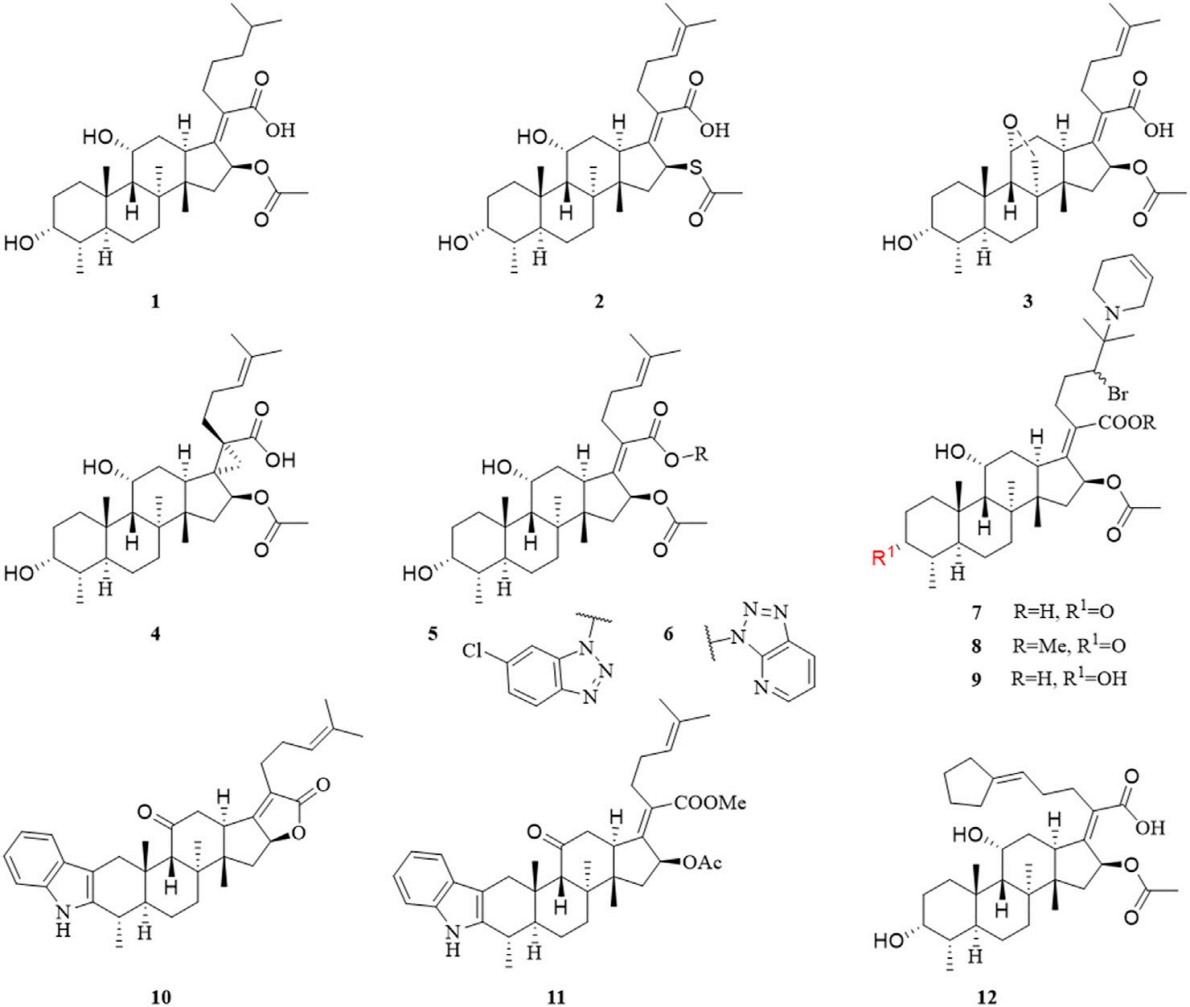
Structural formulae of compounds **1–12**.

Duvold et al. saturated the delta-17 (20) double bond of FA and obtained four stereoisomers, of which only 17(*S*),20(*S*)-dihydro-FA had the same potency as natural FA. This result indicated the necessity for the correct orientation and conformation of the side chains in a limited bioactive space for antimicrobial activity (Duvold et al., 2001). Subsequently, this group introduced a spiro-cyclopropane system in the delta-17 (20) double bond, and successfully synthesized 17(*S*),20(*S*)-methano-FA (**4**) (Figure 2), which exhibited the same activity against several Gram-positive bacteria as FA. This result further showed the importance of the side chains of FA for antimicrobial activity (Duvold et al., 2003).

In 2006, to clarify the interaction between FA and its receptor EF-G, Riber et al. developed three photoaffinity-labeled FA derivatives with the minimum inhibitory concentration (MIC) values of 0.016–4 μg/ml. (Riber et al., 2006). In 2007, Schou et al. have synthesized two radiolabeled photolabile FA analogs. These derivatives are potential tools for revealing the interaction between FA and EF-G (Schou et al., 2007).

In 2018, Salimova et al. synthesized some cyanoethyl derivatives of FA, which were screened primarily *in vitro*. Modification of FA with cyanoethyl fragments did not increase the activity, which is consistent with the previously summarized SAR (Salimova et al., 2018). Lu et al. have designed and synthesized 14 derivatives that blocked the metabolic sites (3-OH and 21-COOH) of FA, six of which had good antibacterial activity, MIC values of compounds **5** and **6** were less than 0.25 μg/ml; however, this result was contrary to previously SAR studies of the 21-COOH, as summarized by Daehne et al. Pharmacokinetic experiments were also performed, compounds **5** and **6** released FA *in vivo*, and their half-life was longer than that of FA. These derivatives provided a new concept for the structural modification of FA, with a triazole ring introduced at the 21-COOH. The activity of these FA derivatives was maintained, indicating that this was a new route to obtain long-lasting and effective antibiotics by structural modification (Daehne et al., 1979; Lu et al., 2019).

Shakurova et al. synthesized three quaternary pyridinium salts and tetrahydropyridine derivatives (**7**, **8**, and **9**) of FA using an effective one-pot method, but after antimicrobial screening, the results showed that there was no inhibitory activity against the tested strains when the concentration of the derivatives was 32 μg/ml (Shakurova et al., 2019). In 2020, Salimova et al. synthesized two new indole derivatives (**10** and **11**) of FA by the Fischer reaction. The antimicrobial activity of the derivatives was tested against MRSA (strain ATCC 43300), and the compounds showed comparable activity to FA (Salimova et al., 2020).

Chavez et al. have synthesized 14 FA analogs, compound **12** has equivalent potency against clinical isolates of *Staphylococcus aureus* and *Enterococcus faecium* as well as an improved resistance profile *in vitro* when compared to FA. Significantly, **12** displays efficacy against FA-resistant strain of *Staphylococcus aureus* in a soft-tissue murine infection model. This study indicated the structural features of FA necessary for potent antibiotic activity and demonstrates that the resistance profile can be improved for this target and scaffold (Chavez et al., 2021).

**FIGURE 3:**
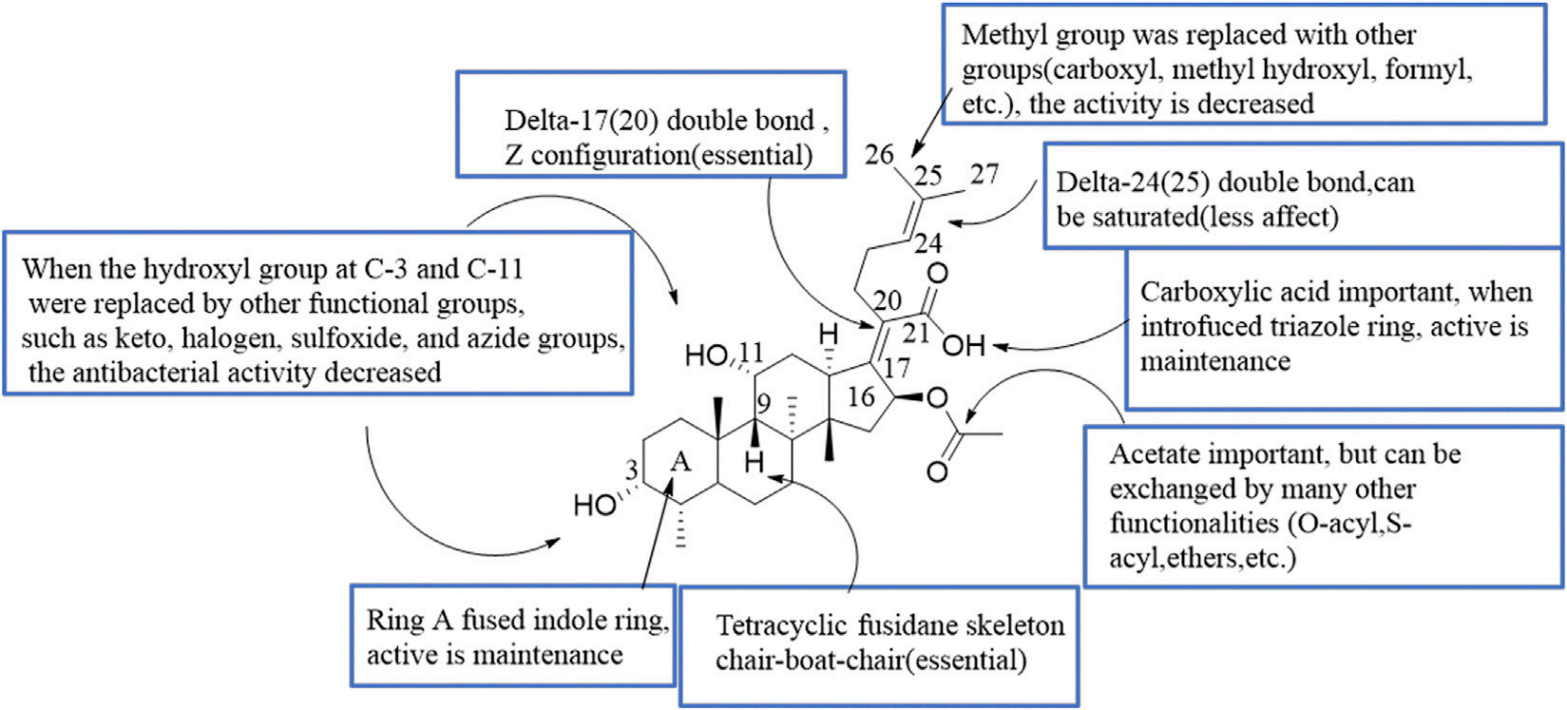
SAR of the antibacterial activity of FA.

Since the marketing of FA, various structural modifications have been made, but only one derivative, l6-deacetoxy-l6*β*-acetylthio FA, is significantly more active than the parent antibiotic (Daehne et al., 1979). This review summarized the SAR of the antimicrobial activity of FA (Figure 3).

Recently, Hajikhani et al. used several international databases to discern studies addressing the prevalence of FA resistant *S. aureus* (FRSA), FA resistant MRSA (FRMRSA), and FA resistant methicillin-susceptible *S. aureus* (FRMSSA). The analyses manifested that the global prevalence of FRSA, FRMRSA, and FRMSSA was 0.5, 2.6, and 6.7%, respectively. These results indicated the need for prudent prescription of FA to stop or diminish the incidence of FA resistance. (Hajikhani et al., 2021).

In conclusion, since the antibacterial activity of FA was found, many structural modifications have been made to FA. However, the antibacterial activity of only two compounds reached the level of FA activity, the antibacterial activity of other derivatives is worse than FA. At present, the SAR summarized according to the existing literature is not perfect and needs to be further enriched. In recent years, drug-resistant bacterial of FA has appeared. It is necessary to study FA derivatives with better activity against drug-resistant bacterial.

#### 2.1.2 Anti-*M. tuberculosis* Activity

According to the WHO, tuberculosis remains the world’s deadliest infectious killer. Worldwide, more than 4,000 people die of tuberculosis every day, and nearly 30,000 people are affected by this preventable and curable disease (World Health Organization, 2020b). In 1962, Godtfredsen et al. studied the antibacterial spectrum of FA and found that FA had some antituberculosis activity, but there was no further research performed (Godtfredsen WO. et al., 1962). In 1990, Hoffner et al. found that FA was effective against 30 clinically isolated *Mycobacterium tuberculosis* (*M. tuberculosis*) strains *in vitro* at concentrations of 32–64 mg/L, and was synergistic with ethambutol against *M. tuberculosis* (Hoffner et al., 1990). Fuursted et al. used a variety of tuberculosis bacilli (including drug-resistant tuberculosis bacilli) and determined the MIC values of FA against tuberculosis bacilli, which ranged from 8 to 32 mg/L (Fuursted et al., 1992; Fabry et al., 1996). Unlike the experimental results of Hoffner, Öztas did not observe either a synergistic or antagonistic effect when FA was used in combination with other standard antituberculosis drugs (Fuursted et al., 1992). The reason for this difference may be because the groups used different test methods. Previous studies have also found that FA was not cross-resistant with first-line drugs (Hoffner et al., 1990; Fuursted et al., 1992).

In 2008, Öztas et al. conducted susceptibility tests for FA in the sputum cultures of 728 tuberculosis patients. The results indicated that FA was effective at 32 mg/L *in vitro*, but resistance to FA was observed at 16 mg/L. This group suggested that FA might be an alternative antituberculosis drug (Öztas et al., 2008). FA was found to lack *in vivo* activity at doses of up to 200 mg/kg in a mouse model of tuberculosis (Shanika, 2017).

In 2014, to solve the problem that FA has no antituberculosis activity *in vivo*, Kigondu et al. adopted a repositioning strategy to determine whether FA could be used as an optional antituberculosis drug. They hope to synthesize and screen FA derivatives to study the antituberculosis activity and mechanism of action (Kigondu et al., 2014). In a recent study, Akinpelu et al. found that FA was a potential inhibitor of *M. tuberculosis* filamentous temperature sensitive mutant Z (FtsZ) by computer methods, including density function theory (DFT), molecular docking, and molecular dynamics simulations (Akinpelu et al., 2020).

**FIGURE 4:**
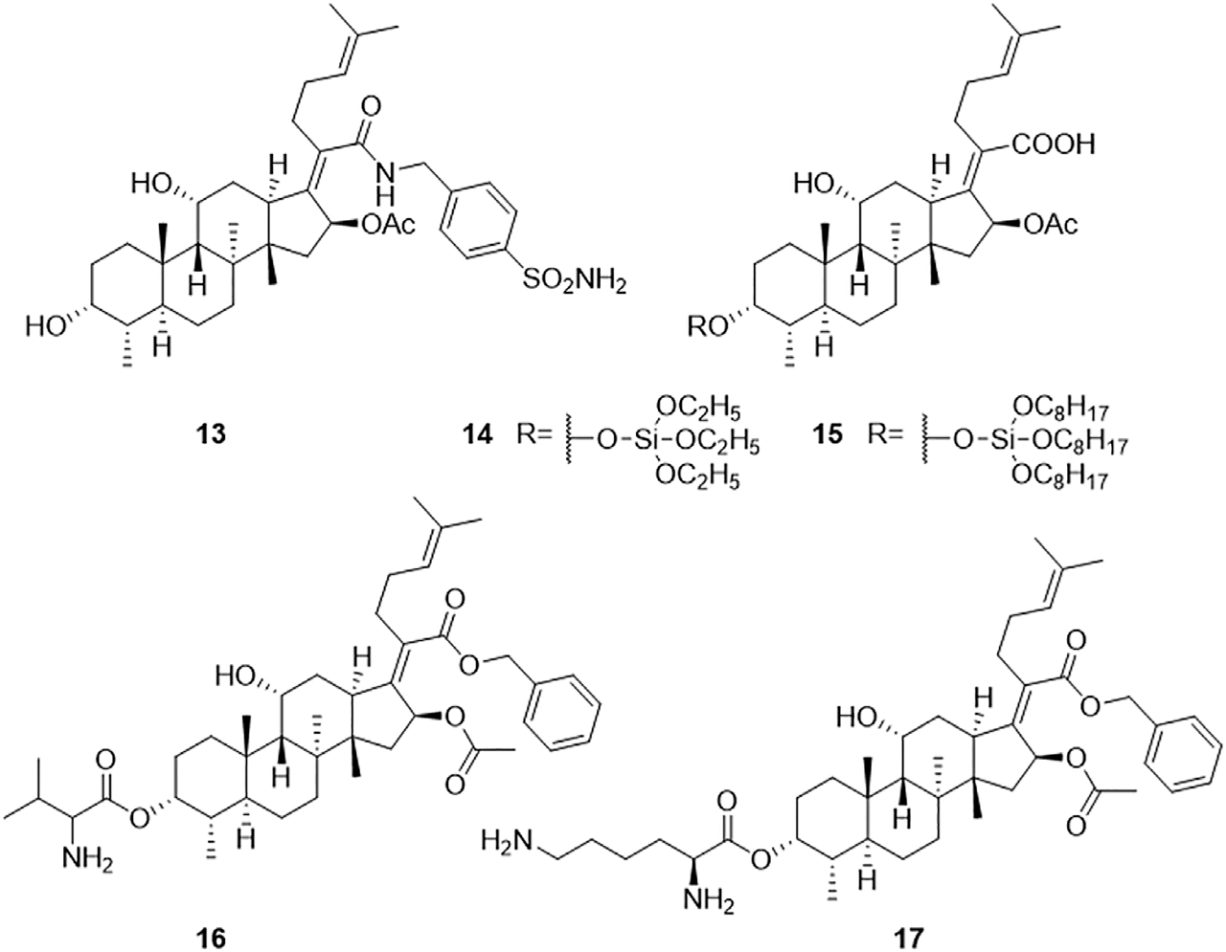
Structural formulae of compounds **13–17**.

Dziwornu et al. have synthesized 28 FA derivatives, which were amidated at the 21-COOH, including C-21 FA ethanamides, anilides, and benzyl amides. All the derivatives were evaluated for their antituberculosis activity using the H37RvMa strain and the minimum inhibitory concentration required to inhibit the growth of 90% of the bacterial population (MIC_90_) values were determined. Compound **13** had the most potent antituberculosis activity with a MIC_90_ value of 2.71 μM, but not as good as FA with a MIC_90_ value of 0.24 μM (Figure 4) (Dziwornu et al., 2019).

Njoroge et al. synthesized 27 derivatives of FA by esterification at the 3-OH and 21-COOH, including C-3 alkyl, aryl, and silicate esters, and the Mtb H37RvMa strain was used to determine the antituberculosis activity of the derivatives *in vitro*. The activities of the C-3 silicate derivatives were similar to that of FA. The minimum concentration required to inhibit the growth of 99% of the bacterial population (MIC_99_) values of compounds **14** and **15** against the Mtb H37RvMa strain were 0.2 and 0.3 μM, respectively, while FA with a MIC_99_ value of <0.15 μM (Njoroge et al., 2019).

Singh et al. used chemical biology and genetics, showed essentiality of its encoding gene fusA1 in *M. tuberculosis* by demonstrating that the transcriptional silencing of fusA1 is bactericidal *in vitro* and in macrophages. Thus, this study identified EF-G as the target of FA in *M. tuberculosis*. (Singh et al., 2021).

**FIGURE 5:**
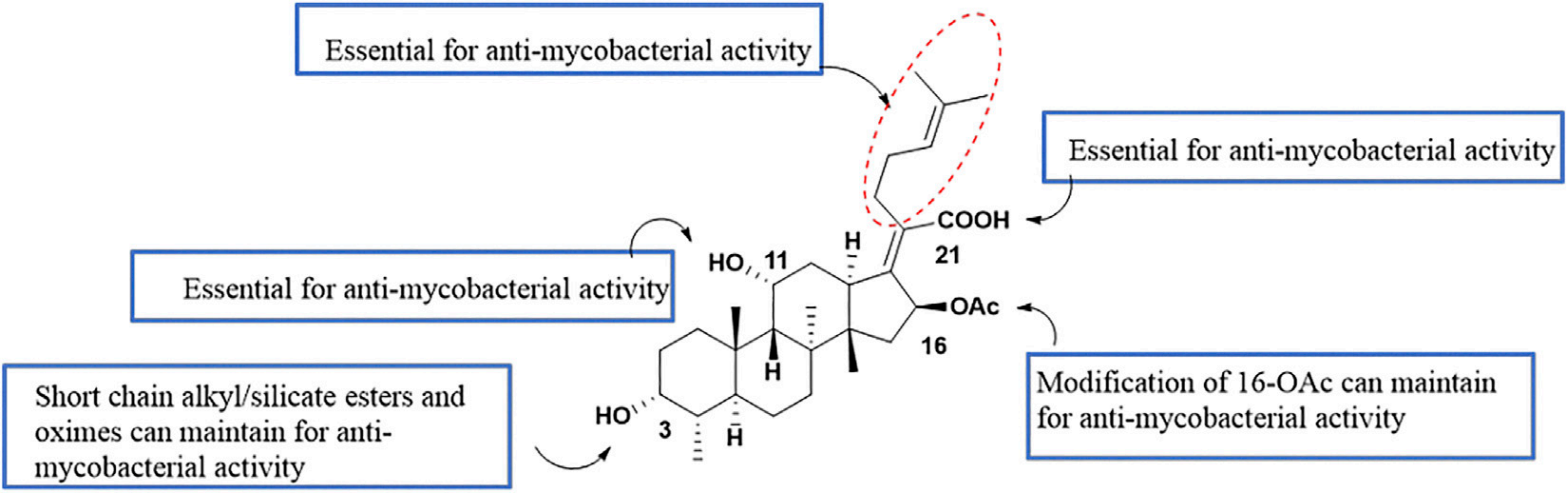
SAR of the antituberculosis activity of FA.

Singh et al. have summarized the preliminary SAR of 58 antituberculosis FA derivatives (Figure 5). It was found that the 11-OH, 21-COOH, and lipid side chains were necessary for antituberculosis activity, while modification at the 3-OH with short chain alkyl or silicate esters and oximes could maintain the activity, and replacing the acetoxy group of C-16 with a propionyloxy group maintained the activity (Singh et al., 2020).

In conclusion, similar to the antibacterial modification of FA, the antituberculosis modification of FA has not made significant progress and needs to be further explored. And the reason why FA has no antituberculosis activity *in vivo* needs to be further clarified. It is also necessary to continue to study FA derivatives with antituberculosis activity *in vivo* and *in vitro*. If the above problems are solved, FA will be repositioned as an antituberculosis drug with novel mechanism of action.

#### 2.1.3 Antifungal Activity

Many adults and pediatric patients use strong chemotherapy agents to treat hematological malignancies, thus increasing the incidence of invasive mycosis (Zając-Spychała et al., 2019; Malhotra, 2020). Because antifungal drugs are available in a limited number and are prone to drug resistance, there is a view that the key to the future development of antifungal drugs is the repurposing of marketed drugs (Zida et al., 2017; Nicola et al., 2019).

FA itself has no antifungal activity, but recently it has been reported that FA derivatives have antifungal activity. Cao et al. inadvertently found that FA derivative **16** inhibited the growth of *Cryptococcus neoformans*. The inhibition rate of compound **16** against *C. neoformans* was 94.58% at a concentration of 32 μg/ml. Among the reported compounds, compound **17** had the strongest MIC value (4 μg/ml) against *C. neoformans* (Cao et al., 2020). In another study, Shakurova et al. synthesized quaternary pyridinium salts, and the tetrahydropyridine derivative **9** had moderate activity at a concentration of 32 μg/ml against *C. neoformans* (Figure 4) (Shakurova et al., 2019).

There are a limited number of antifungal FA derivatives reported in the literature, but the data provide insights for the development of FA antifungal activity. Furthermore, this information provides guidance for the future design of FA derivatives with good antifungal activity and selectivity.

### 2.2 Antiparasitic Activity

#### 2.2.1 Antimalarial Activity

According to the World Health Organization (WHO) World Malaria Report 2020, it was estimated that there were 229 million new malaria infections, and 409,000 people died of malaria, worldwide in 2019 (World Health Organization, 2020a). *Plasmodium falciparum* is resistant to existing antimalarial drugs, including artemisinin, which poses a challenge for antimalarial treatment. Therefore, there is an urgent need for new antimalarial drugs, especially those with novel mechanisms of action and no cross-resistance to existing drugs (Biddau and Sheiner, 2019).

As early as 1985, FA was found to have antimalarial activity *in vitro* (Black et al., 1985). Johnson et al. found that FA killed malaria parasites (*P. falciparum* line D10) with an IC_50_ value of 52.8 µM, and then characterized the possible target of FA against malaria, which is EF-G in two organelles of *Plasmodium*, the apicoplast and mitochondria. It could be an effective lead compound because of its mechanism of action (Johnson et al., 2011). Compared with *P. falciparum* mitochondria EF-G, FA had a better effect on apicoplast EF-G. The reason for mitochondrial EF-G resistance is at least partly because there is a conservative three amino acid sequence (GVG motif) in the switch I loop, however this motif is not found in apicoplast EF-G (Gupta et al., 2013).

**FIGURE 6:**
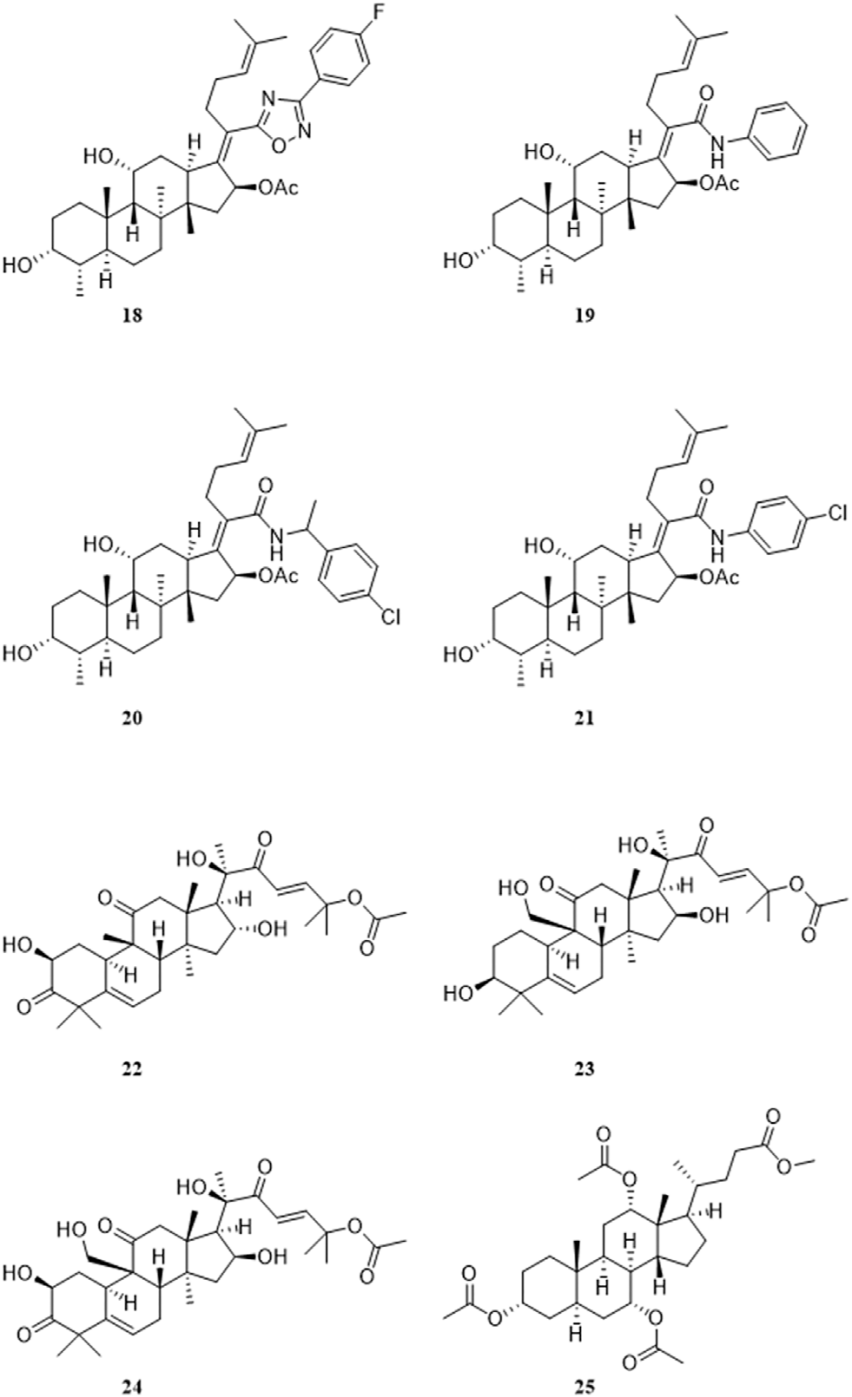
Structural formulas of compounds **18–25**.

Kaur et al. synthesized a series of compounds in which the 21-COOH of FA was substituted with various bioisosteres, and evaluated the activity *in vitro* with the chloroquine-sensitive NF54 strain of the malaria parasite *P. falciparum*. Among these compounds, the antiplasmodial activity IC_50_, CC_50_ and selection index of the most active compound **18** were 1.7, 77.4, and 46 μM, respectively. The IC_50_, CC_50_, and selection index of FA were 59.0, 194.0, and 3 μM, respectively. Compared with FA, compound 18 has a higher SI value. Furthermore, this group constructed apicoplast and mitochondrial EF-G homology structure models of *P. falciparum*, and compound **18** was docked with these two models. The docking results showed that the EF-G binding site of compound **18** and FA was consistent, but compound **18** had a higher binding score (Figure 6) (Kaur et al., 2015).

Espinoza-Moraga et al. amidated or esterified the 21-COOH in FA with various substituents, including ester chains and aromatic compounds, and evaluated the antiplasmodial activity of these compounds *in vitro* against the chloroquine-sensitive NF54 strains and multidrug-resistant K1 strains of the malarial parasite *P. falciparum*. Compound **19** had the best antiplasmodial activity, with IC_50_ values of 1.2 and 1.4 μM against the NF54 and K1 strains, respectively. Unfortunately, the mechanism of action was not explored (Espinoza-Moraga et al., 2016). Kaur et al. developed a 3D-QSAR model based on the antiplasmodial activity of 61 FA derivatives that they had synthesized previously. The verified Hypo2 model was used as a three-dimensional structure search query to screen combinatorial libraries based on FA. Eight virtual screening hit compounds were selected and synthesized, of which compounds **20** and **21** had IC_50_ values of 0.3 and 0.7 μM, respectively, for the NF54 strain of *P. falciparum*. The IC_50_ values of these two compounds for the drug-resistant K1 strain of *P. falciparum* were both 0.2 μM, and no appreciable cytotoxicity was detected (Kaur et al., 2018).

Pavadai et al. used FA as a search query, and adopted two-dimensional fingerprint- and three-dimensional shape-based virtual screening methods to obtain new inhibitors of *P. falciparum* from their in-house database, including 708 steroid-type natural products. After further screening, this group successfully identified nine compounds that inhibited the growth of the NF54 strain of *P. falciparum*, with IC_50_ values of less than 20 μM. The IC_50_ values of the four most active compounds **22–25** were 1.39, 1.76, 2.92, and 3.45 μM, respectively. Moreover, the predicted absorption, distribution, metabolism, and excretion (ADME) properties of these four compounds were comparable to FA (Pavadai et al., 2017).

To date, the chemical modification of FA for antimalarial activity has been mainly concentrated on the 21-COOH. Esterification or amidation is beneficial to the activity, and amidation is better than esterification for activity. The structural modification of other sites of FA needs to be further explored. FA derivatives have good inhibitory activity against *P. falciparum*, and apicoplast EF-G is the main action site of FA. It is necessary to modify FA to improve selectivity for apicoplast EF-G. Thus, FA derivatives have potential to be repositioned as an antimalarial drug.

#### 2.2.2 Other Antiparasitic Activities

Rizk et al. described the inhibitory effects of FA on the *in vitro* growth of bovine and equine *Babesia* and *Theileria* parasites. The *in vitro* growth of four *Babesia* species that was significantly inhibited by micromolar concentrations of FA (IC_50_ values = 144.8, 17.3, 33.3, and 56.25 µM for *Babesia bovis*, *Babesia bigemina*, *Babesia caballi*, and *Theileria equi*, respectively). These results indicate that FA might be incorporated in treatment of *babesiosis* (Rizk et al., 2020).

Payne et al. investigated the therapeutic value of FA for *T. gondii* and found that the drug was effective in tissue culture, but not in a mouse model of infection. This work highlights the necessity of *in vivo* follow-up studies to validate *in vitro* drug investigations. (Payne et al., 2013).

### 2.3 Tumor Related Activity

#### 2.3.1 Antitumor Activity

**FIGURE 7:**
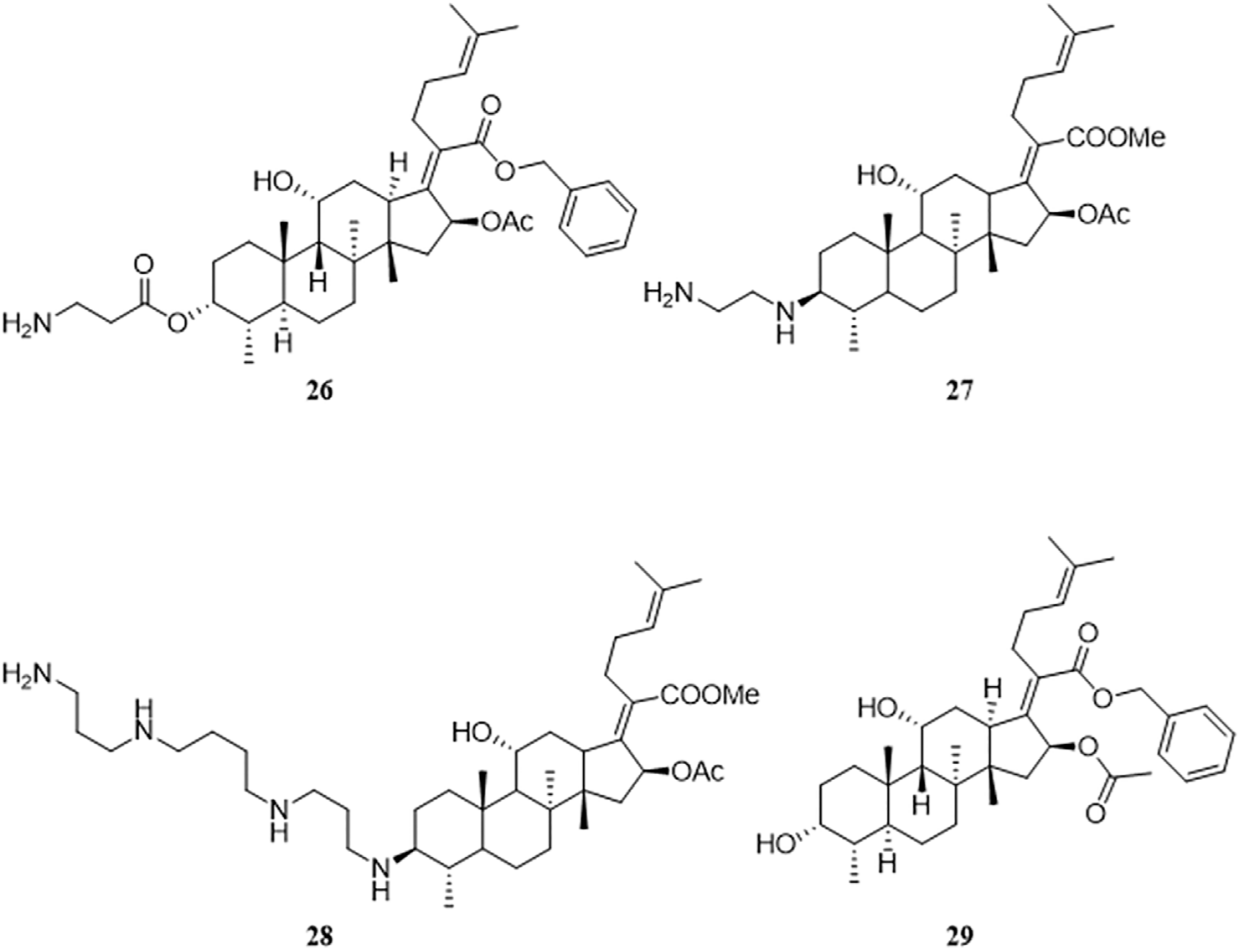
Structural formulae of compounds **26–29**.

Malignant tumors are a health issue all over the world. There were an estimated 19.3 million new cases of cancer and almost 10.0 million deaths from cancer worldwide in 2020. (Ferlay et al., 2021). In 2019, Ni et al. accidentally discovered that FA derivatives have antitumor activity. Among the derivatives synthesized by this group, compounds where the 21-COOH was modified by a benzyl group, and with amino terminal modification at the C-3 position, had antitumor activity, of which compound **26** was the most active compound (Ni et al., 2019). Compound **26** had antitumor activity against various tumor cell lines including HeLa, U87, KBV, MKN45, and JHH-7, with IC_50_ values ranging from 1.26 to 3.57 μM. A preliminary mechanistic study was performed, which indicated that neo-synthesized proteins were decreased in HeLa cells under the action of compound **26**, and the ratio of cells in the Sub-G_0_/G_1_ phase was increased, as determined by flow cytometry monitoring, thus leading to HeLa cell apoptosis. Compound **26** also exhibited good antitumor activity *in vivo* against a xenograft tumor of HeLa cells in athymic nude mice (Figure 7).

Salimova et al. adopted different substituted amino groups to modify the 3-OH of FA, with or without esterification of the 21-COOH, to synthesize a series of 3-amino-substituted FA derivatives. To determine the antitumor activity of these derivatives, the researchers used nine different types of human tumor cell lines (sourced from the American Cancer Institute NCI-60) to study the antitumor activity of these compounds *in vitro*. Compound **27** had the highest cytotoxicity against leukemia cells and compound **28** had the broadest antitumor activity, including against leukemia, non-small cell lung cancer, colon cancer, neurological tumors, melanoma, ovarian cancer, and renal cancer (Salimova et al., 2019).

**FIGURE 8:**
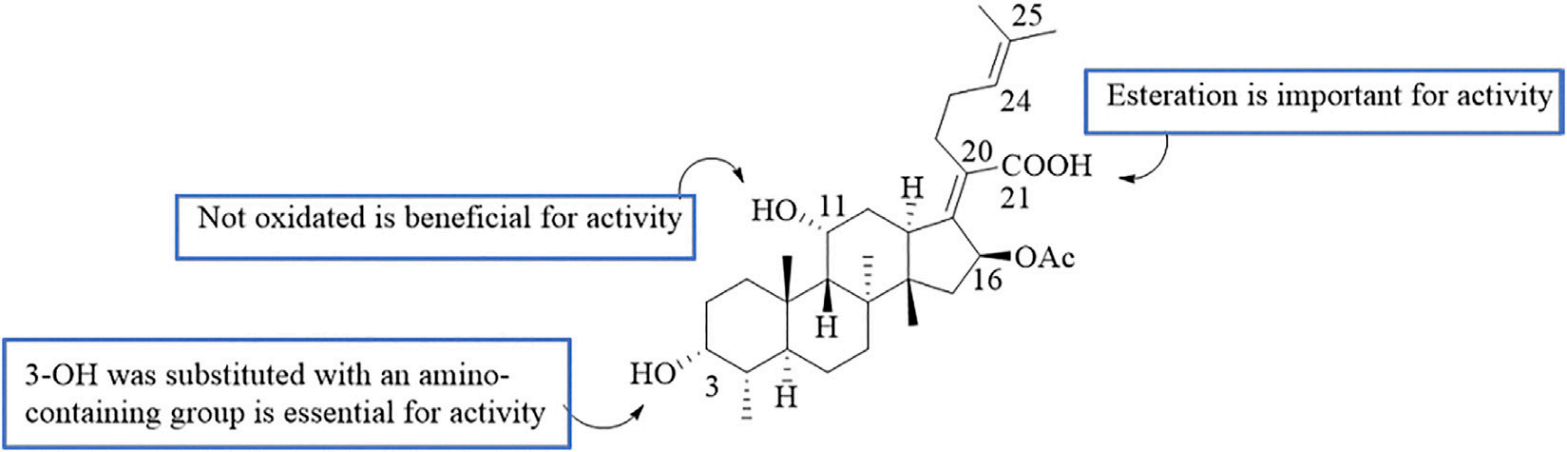
SAR of the antitumor activity of FA.

By analyzing the relationship between antitumor activity and structure of FA, the following preliminary SAR was obtained (Figure 8) (Ni et al., 2019; Salimova et al., 2019).

#### 2.3.2 Tumor Multidrug Resistance Reversal Activity

MDR is the main cause of drug resistance in many tumors, and is the main factor leading to the failure of chemotherapy. MDR affects patients with a variety of hematological and solid tumors (Persidis, 1999). Drug-sensitive cells can be killed by chemotherapeutics, but there may be a proportion of drug-resistant tumor cells left behind, which grow later, resulting in resistance to chemotherapeutics, leading to treatment failure (Lage, 2008). Currently, it is considered that the most effective strategy to overcome MDR is to develop MDR reversal agents.

Several MDR reversal agents have failed in clinical trials because of inherent toxicity, low selectivity, or complex pharmacokinetic interactions (Palmeira et al., 2012). Therefore, a safe and effective MDR reversal agent with low toxicity is urgently needed. To date, many natural products with different structural types have been developed as potential MDR reversal agents (Kumar and Jaitak, 2019).

Guo et al. found that FA derivatives have tumor MDR reversal activity. The derivative **29**, which was modified with a benzyl group at the 21-COOH, had good MDR reversal activity *in vitro*. Further studies revealed that the combination of derivative **29** with paclitaxel re-sensitized the multidrug-resistant oral epidermoid carcinoma (KBV) cell line to paclitaxel. A mechanism study found that compound **29** enhanced the ATPase activity of P-glycoprotein (P-gp) by inhibiting the drug pump activity of P-gp, but did not affect the expression of P-gp (Guo et al., 2019).

**FIGURE 9:**
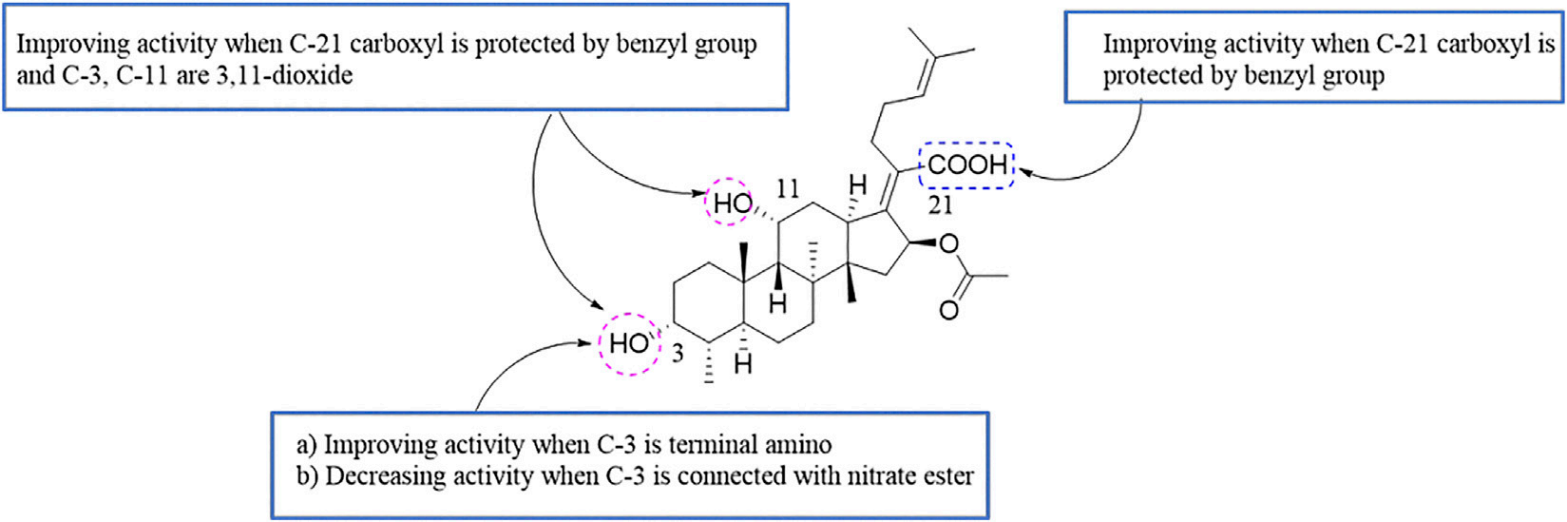
SAR of the tumor multidrug resistance reversal activity of FA.

According to the results for MDR reversal activity, the SAR of FA derivatives has been preliminarily summarized (Figure 9).

According to the existing literature, FA has certain potential in antitumor and tumor MDR reversal activity. However, there are few studies in antitumor and tumor MDR reversal activity of FA, which needs to be further explored.

### 2.4 Anti-Inflammatory Activity

Inflammation is a complex biological response to injury, and to attack pathogens as part of the body’s immune response, which results in symptoms that include pain, fever, erythema, and edema (Ferrero-Miliani et al., 2007). The impact of antimicrobial agents on the immune and inflammatory systems and their possible clinical significance have greatly attracted the interest of scientists (Bosnar et al., 2019).

**FIGURE 10:**
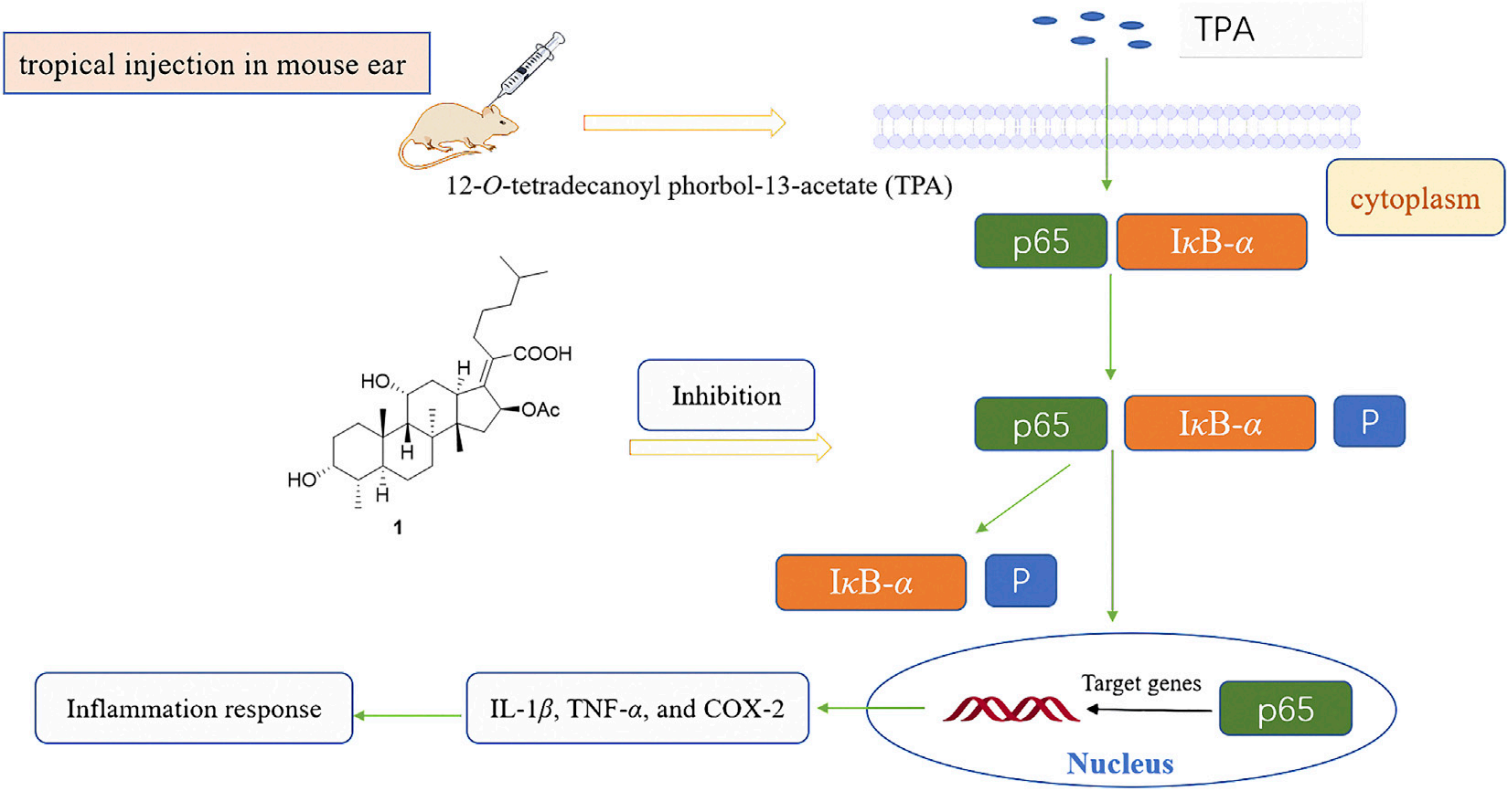
Schematic representation of the anti-inflammation mechanism of compound **1** in TPA-induced mouse ear edema models.

FA has been found to have some anti-inflammatory effects in mice and rats *in vivo*, especially by reducing the release of tumor necrosis factor alpha (TNF-*α*) (Table 1) (Bosnar et al., 2019). In 2018, Wu et al. saturated the delta-24 (25) double bond of FA and thus obtained the hydrogenated derivative **1**. The antimicrobial activity of FA and compound **1** were tested against six bacterial strains, and the results showed that both FA and **1** showed high levels of antimicrobial activity against Gram-positive strains. The anti-inflammatory activity of these compounds was evaluated using the 12-*O*-tetradecanoyl phorbol-13-acetate (TPA)-induced mouse ear edema model. The results showed that FA and **1** effectively reduced TPA-induced ear edema in a dose-dependent manner, and this inhibitory effect was associated with the inhibition of TPA-induced upregulation of the pro-inflammatory cytokines IL-1*β*, TNF-*α*, and COX-2. Furthermore, **1** significantly inhibited the expression levels of p65, I*κ*B-*α*, and *p*-I*κ*B-*α* in TPA-induced mouse ear edema models (Figure 10) (Wu PP. et al., 2018).

**TABLE 1:** Experiments show that FA modulates immunity and the inflammatory process.

Pharmacological actions	References
FA decreased the plasma peak of TNF-α, improved the survival rate of neonatal mice, and decreased plasma TNF-α during endotoxic shock	Genovese et al. (1996)
FA protected mice from concanavalin-A-induced hepatitis. At the same time, the plasma levels of IL-2, IFN-γ, and TNF-α were significantly decreased, but the levels of IL-6 were increased	Nicoletti et al. (1998)
FA was beneficial for the treatment of experimental autoimmune neuritis in rats (a model of Guillain-Barre syndrome), where the serum levels of interferon-gamma, IL-10, and TNF-α were reduced	Marco et al. (1999)
FA could alleviate the tissue edema caused by local formalin injection in rats	Kilic et al. (2002)
The co-administration of FA and daptomycin significantly reduced the joint and tissue levels of systemic TNF-α, IL-6, IL-1β, and other pro-inflammatory cytokines in mice infected with multidrug-resistant group B streptococci	El-Shemi and Faidah (2011)

According to current research, FA derivative has showed anti-inflammatory activity *in vivo* and *in vitro*. However, there is no literature yet reported the structural modification of anti-inflammatory of FA, which needs to be enriched. And other possible anti-inflammatory mechanisms need to be further studied.

### 2.5 Antiviral Activity

Severe Acute Respiratory Syndrome Coronavirus 2 (SARS-CoV-2), a new type of RNAβ coronavirus, has caused a pandemic worldwide (Gallelli et al., 2020). There is currently no effective treatment for the virus, and effective preventive and therapeutic drugs need to be identified (Sanders et al., 2020). There have been many reports of antibiotic agents that have antiviral activity. Minocycline, a tetracycline drug, can effectively inhibit human immunodeficiency viruses (HIV) (Zink et al., 2005). Aminoglycoside antibiotics can inhibit the replication of the herpes simplex virus, influenza A virus, and Zika virus *in vivo* and *in vitro* (Gopinath et al., 2018).

As early as 1967, the first antiviral research on FA appeared, FA was found to be ineffective against coxsackie A21 or rhinovirus infection, both orally and intranasally, and fifteen derivatives were found to be inactive or toxic (Acornley et al., 1967). There have been several reports regarding the effectiveness of FA toward human immunodeficiency virus (HIV), and the mechanism of action has also been studied. FA is an anionic surfactant that acts on the lipid molecular layer of infected cells, exposing the viral proteins to the host immune system to prevent the HIV from forming syncytium *in vitro* (Lloyd et al., 1988). Additionally, FA can directly inhibit reverse transcriptase and thus has anti-HIV activity (Famularo et al., 1993). Four clinical trials of FA in HIV-infected patients have been conducted, but the results were contradictory (Faber et al., 1987; Youle et al., 1989; Hørding et al., 1990; Famularo et al., 1993). Furthermore, a study has shown that human leukocyte interferon can enhance the anti-HIV effect of FA (Degre and Beck, 1994).

In recent years, it has been reported that FA was effective against John Cunningham virus (JCV) *in vivo* and *in vitro* (Brickelmaier et al., 2009; Chan et al., 2015). Liu et al. found that FA had good antiviral activity against enterovirus A71 (EV-A71) and coxsackievirus A16 (CV-A16). The potential antiviral mechanism is related to the inhibition of viral RNA replication and the synthesis of viral proteins (Liu et al., 2019). Kwofie et al. found that FA was the potential anti-SARS-CoV-2 compounds by antiviral activity predictions (Kwofie et al., 2021).

Although it has been found that FA has good *in vivo* and *in vitro* activity against a variety of viruses, there have been few studies on the antiviral effects of FA derivatives, which merit future exploration. Furthermore, whether FA has a therapeutic effect against SARS-CoV-2 is also a possible research direction.

### 2.6 Other Activities

Some antibiotics have been discovered to have the potential to treat neurological diseases, acting as neuroprotective agents via various pathways, including rifampicin, rapamycin, d-cycloserine, and ceftriaxone (Batson et al., 2017; Lin et al., 2017; Reglodi et al., 2017; Wu X. et al., 2018). Additionally, the property of easily crossing the blood-brain barrier is one of the necessary criteria for a neuroprotective agent, and researchers have found that FA has this characteristic (Mindermann et al., 1993).

Park et al. first discovered that FA had neuroprotective effects. This group used sodium nitroprusside (SNP) to pretreat C6 glial cells, and found that FA prevented SNP-induced cell death in a dose-dependent manner at 5–20 μM. Moreover, a mechanism study was performed, and the results indicated that FA had a neuroprotective effect against SNP-induced cytotoxicity through the 5′ adenosine monophosphate-activated protein kinase (AMPK) pathway and apoptotic events (Park et al., 2019).

Unfortunately, this research was only performed *in vitro*, not *in vivo*. Additionally, the mechanism of action of FA was not fully elucidated, and more studies are needed to clarify the mechanism. Nevertheless, the results of this study have provided a potential clinical strategy, suggesting that FA derivatives may be used for the treatment of neurological disease as neuroprotective agents.

## 3 Conclusion and Perspectives

FA can be obtained by fermentation, and has attracted increasing attention in recent years. In summary, FA is a promising natural bioactive substance with a variety of pharmacological activities for the potential treatment of many diseases. Great progress has been achieved in the investigation of the pharmacological activity, SAR, and mechanism of action of FA. FA consists of a tetracyclic skeleton with several available sites for chemical modification, which enables the synthesis of novel compounds with potentially higher potency and selectivity, and with fewer side effects.

Despite extensive research and development into FA in recent years, considerable challenges still lie ahead because of the limited amount of studies on the pharmacological activities and mechanisms of action to date. FA has a very short half-life after oral absorption. Consequently, FA must be administered frequently, resulting in fluctuations in the plasma drug concentration and increasing the risk of poor clinical outcomes, including side effects and adverse reactions, limiting the application of FA in clinical use. Considering that triterpenes are known to possess a wide range of pharmacological activities, it is possible that other new pharmacological effects of FA still to be discovered. Much of the present research has been confined to *in vitro* rather than *in vivo* studies; hence, whether FA is effective or sufficiently efficient *in vivo* is questionable and must be validated.

In view of the above challenges, the following strategies will be of great value in future research into the drug development and clinical application of FA:

1) Extending the half-life of FA by adopting appropriate pharmaceutic or chemical methods. For example, FA is administered in liposomes or structural modifications that occlude the 21 COOH metabolic site.
2) From the view of the pharmacology and mechanism, further investigation of the potential pharmacological activities of FA expands the scope of its use. Meanwhile, more research into the mechanism of action will enable a better understanding of how FA works. Furthermore, a large number of *in vivo* studies should be conducted to validate its effectiveness, because a high sensitivity *in vitro* study does not necessarily represent the same result *in vivo*.
3) Synthesizing novel derivatives by structural modification at the confirmed modification sites, or other potentially available sites of FA, to explore more promising agents with higher activity and better drug-like properties.
4) As a clinically used drug, FA has the possibility of repositioning as an antituberculous or antimalarial drug, which requires more research in these fields. The antitumor, tumor MDR reversal, anti-inflammatory, antifungal activities of FA are newly discovered biological activities in recent years, which have larger research value.

In conclusion, the knowledge regarding FA has been growing rapidly in recent years, but there is still room for improvement in the understanding of its pharmacology, mechanism of action, and structural modification.
